# An investigation into pharmaceutically relevant mutagenicity data and the influence on Ames predictive potential

**DOI:** 10.1186/1758-2946-3-51

**Published:** 2011-11-22

**Authors:** Patrick McCarren, Clayton Springer, Lewis Whitehead

**Affiliations:** 1 Novartis Institutes for Biomedical Research, 100 Technology Square, Cambridge, MA 02139, USA

## Abstract

**Background:**

In drug discovery, a positive Ames test for bacterial mutation presents a significant hurdle to advancing a drug to clinical trials. In a previous paper, we discussed success in predicting the genotoxicity of reagent-sized aryl-amines (ArNH_2_), a structure frequently found in marketed drugs and in drug discovery, using quantum mechanics calculations of the energy required to generate the DNA-reactive nitrenium intermediate (ArNH:+). In this paper we approach the question of what molecular descriptors could improve these predictions and whether external data sets are appropriate for further training.

**Results:**

In trying to extend and improve this model beyond this quantum mechanical reaction energy, we faced considerable difficulty, which was surprising considering the long history and success of QSAR model development for this test. Other quantum mechanics descriptors were compared to this reaction energy including AM1 semi-empirical orbital energies, nitrenium formation with alternative leaving groups, nitrenium charge, and aryl-amine anion formation energy. Nitrenium formation energy, regardless of the starting species, was found to be the most useful single descriptor. External sets used in other QSAR investigations did not present the same difficulty using the same methods and descriptors. When considering all substructures rather than just aryl-amines, we also noted a significantly lower performance for the Novartis set. The performance gap between Novartis and external sets persists across different descriptors and learning methods. The profiles of the Novartis and external data are significantly different both in aryl-amines and considering all substructures. The Novartis and external data sets are easily separated in an unsupervised clustering using chemical fingerprints. The chemical differences are discussed and visualized using Kohonen Self-Organizing Maps trained on chemical fingerprints, mutagenic substructure prevalence, and molecular weight.

**Conclusions:**

Despite extensive work in the area of predicting this particular toxicity, work in designing and publishing more relevant test sets for compounds relevant to drug discovery is still necessary. This work also shows that great care must be taken in using QSAR models to replace experimental evidence. When considering all substructures, a random forest model, which can inherently cover distinct neighborhoods, built on Novartis data and previously reported external data provided a suitable model.

## 1. Introduction

### 1.1 Aims

In the field of drug-discovery, a positive Ames test can halt development of a particular chemotype and possibly work on an entire drug target because genotoxicity of a potential therapeutic would be a serious issue that needs to be avoided. Sufficiently nuanced rules do not exist to fix such a problem while maintaining the careful balance of potency and properties. Compounding this problem is that impurities or metabolites that could be generated in parts-per-million quantities (10 μg/day) are just as serious from a regulatory standpoint, which could eliminate an essential core structure. Thus, prediction of whether a starting material, degradation product, or drug will be mutagenic in the Ames genotoxicity test is our primary goal. More specifically, our initial focus was on aryl-amines, which are commonly used reagent building blocks in many small molecule drug-discovery projects and appear as a substructure in at least 13% of currently marketed drugs [1]. Aryl-amines also have a known mechanism for genotoxicity. In a previous article, we have shown that an *in silico *assessment of aryl-amines using quantum mechanics reaction energy calculations can provide excellent detection of mutagenic aryl-amines [2]. However, we were surprised that statistical models incorporating additional descriptors did not improve the performance of the single nitrenium formation energy parameter given the wealth of QSAR literature showing accuracy approaching or exceeding the known experimental error. Additionally, we found that the set of Novartis aryl-amines was surprisingly challenging to model compared to those in the literature.

Our ultimate goal is to provide medicinal chemists with usable models to improve the chances of avoiding a toxicity trap that is often visible only after low-throughput tests come back. The aryl-amines can be predicted reliably with the nitrenium formation energy calculation but comparing all-substructure external Ames results to our Novartis results, we found that these were also much harder. Other groups in pharmaceutical companies have noted difficulties in predicting mutagenicity in aryl-amines [3], and in internal all-substructure data sets using commercial software [4,5].

Previous to this article, differences between data sets typically used in the literature for building mutagenicity predictive methods and the data at pharmaceutical companies have not been compared. This is key to the disconnect from literature studies and pharmaceutical studies. The high level of performance of statistical models in this arena with constructed test sets is misleading and does not reflect performance in pharmaceutically relevant sets. Here we show the relative difficulty in predicting the Ames test result in the Novartis aryl-amines and other substructures, in contrast to literature sets.

### 1.2 The Significance of the Ames Test

Many compounds in the environment released from industrial pollution and production are known to cause cancer [6]. Regulatory agencies around the world in cooperation with industry experts have adopted stringent test methods to identify and regulate the use of chemical mutagens that might be exposed to the environment or administered to humans directly as pharmaceuticals [7]. Carcinogenicity is usually determined by an array of in-vivo and in-vitro surrogate tests, which are specified by regulatory authorities before administration to man. The Ames bacterial test is a simple experiment to perform and it is a mandatory regulatory test that has been in use for almost 40 years and correlates with life-time rodent carcinogenicity studies that require 2 years to complete [8,9].

At the molecular level, this test for mutagenicity [10,11] detects a substance's ability to cause mutations in engineered strains of Salmonella typhimurium by observing return of function by point mutations in an altered His operon gene. The mutations in the His operon strains prevents histidine biosynthesis, thus random mutations or mutations due to an external agent must occur for colony growth on histidine-deficient medium. Many compounds are converted to mutagenic compounds after metabolism, so the test is performed with and without pre-incubation of the compound with rat liver enzymes. The bacterial strains used in the test have been further engineered to have permeable cell membranes, a reasonably high spontaneous mutation rate, and diminished DNA repair capacity [12].

Although the result of the Ames test can be reported as a standardized quantity of the number of colonies formed, in most recent studies and databases, including the Novartis internal test results, are reported as categorical results: "Ames-positive" (Ames+) or "Ames-negative" (Ames-). Additionally, it has been shown that the qualitative carcinogenicity result is not improved by quantitative mutagenicity potency data [8]. An increase in number of colonies over control by at least a factor of 2 and a clear dose dependence in the mini-Ames screening test [13] is classified as a positive result. Although high-throughput screening assays exist, they do not faithfully predict the result of the Ames test and at the same time require a significant investment [14,15]. Consequently, the volume of data available for the Ames test is fairly limited. The turnaround time and cost for Ames testing makes accurate in silico models quite useful.

There are some limitations of the Ames test that present a challenge to building accurate in silico models. The exact sensitivity of the test for carcinogenicity is somewhat controversial [8], but in a recent retrospective analysis by FDA and EPA researchers of carcinogenicity and surrogate test results showed the Ames test is positive for 49% (275Ames+/557 rodent carcinogens) of carcinogenic compounds but only 19% of the Ames+ compounds are not carcinogenic to rats (85 Ames+/431 rodent non-carcinogens) [16]. Reproducibility both across and inside one laboratory conducting the test is another serious issue. Both literature and internal intra-laboratory assessments of the test, at least in a 2-strain screening version of the test, have found discrepancies on the order of 15-20% [9]. Based on a retrospective analysis of 237 compounds at Novartis with multiple Ames screen test results, this is a realistic estimate; there were 49 (21%) with discrepant results. Among aryl amines, 13 out of 57 compounds with multiple test results were discordant (23%). The test is sensitive and uses high concentrations of the test chemical, which can increase the effect of impurities including metals [17], degradation products, or reagents [18,19]. The chemical can also be toxic to the bacterial system, most notably antibacterials or cytotoxic compounds, but must still be tested to the maximum possible concentration [7].

### 1.3 Substructure alert and QSAR methods

The cause of cancer through the action of chemicals has been studied extensively, and the process typically begins with the chemical, or one of its metabolites, interacting with DNA, which subsequently leads to mutations [20]. The principle of mutagenicity through reaction of DNA with electrophiles has been especially useful in rationalizing and deriving "toxicophores," substructures that are strongly associated with mutagenicity [21,22]. Some of these mechanisms have been studied carefully in vitro and in vivo [23], and DNA or protein adducts can be measured and observed experimentally [24,25]. The first line of defense in avoiding carcinogenicity in drug design is through the use of alerts to chemicals commonly associated with carcinogenicity, mostly derived from environmental testing [22,26]. Kazius et al. provided an analysis of mutagenicity data correlating chemical substructures to mutagenicity [21,27,28]. Most of these toxicophores are associated with Michael acceptors, electrophiles, or enophiles including α,β-unsaturated carbonyl systems, aziridines and epoxides, aliphatic halides, azides, and acid halides. Others such as aryl-amines and nitroaromatics are known to be converted to more reactive species through oxidation, reduction, and conjugation metabolism reactions [29]. The simplest of prediction systems search a chemical for these substructures and uses rules to correctly predict the Ames+ compounds for known mutagenic substructures. However, despite the inclusion of detoxifying rules, these methods misclassify many of the Ames- compounds as positive. For chemical sets containing many unknown classes of mutagens, structural alert systems like DEREK correctly predict only around 50% of the Ames+ compounds [5,30,31]. A similar result (55% sensitivity) was found for compounds tested at Novartis (all mini-Ames screening results, August 2009) using an internally modified DEREK rule set [32]. There is a long history of modeling mutagenicity on chemicals expected to be encountered from environmental and food exposure [9,33-37]. Recent reviews on statistical models of mutagenicity [9,33,38,39] and a recent collaborative head-to-head mutagenicity prediction challenge summarize the current state of the art for external sets [40]. A summary of some recent models is included in the Supporting Information (Additional file 1) which provided an accuracy in test set molecules ranging from 0.73-0.85 (approaching the known error in the experiment) using a number of statistical approaches. A few studies that could be described as using a hybrid approach by identifying the most applicable out of a selection of models have also been developed with extremely good performance [40,41].

## 2. Methods

### 2.1 Data set preparation

Ames test data is available from a number of sources including literature reviews, regulatory agencies, and funding agencies [42]. For our analysis, we focused on an internal Novartis set and two literature sets (combined into one) for aryl-amines and four datasets covering all substructures as detailed in Table 1.

**Table 1:** Aryl-amine mutagenicity data sets considered in this study.

Set	Description	Number of molecules	Number of Ames+	Number of Ames-
Aryl-amines Only				

**A**	Novartis 2009	327	72	255

**B**	Kazius 2005[21]/Hansen 2009[38]	461	327	134

All compound classes				

**C**	Novartis	2754	360	2394

**D**	Hansen[38]	6512	3503	3009

**E**	Marketed Pharmaceuticals[44]	378	44	334

**F**	Kazius[21]^a^	4195	2343	1852

	Combined C, D, E, and F^a^	9423^a^	3591^b^	5832^b^

^a^MW < 700

^b^Ames+ if either C, D, E, or F are Ames+

For the aryl-amine sets, molecules with other substructures associated with mutagenicity, such as nitroaromatic, nitrile oxide, N-nitroso substructures, were removed from the analysis. Set A was from internal Novartis Ames screening test results tested in one laboratory up to 2009. Set B is the aryl-amine subset from compilations published by Hansen et al [38,43] and Kazius et al [21]. All Ames screening results at Novartis excluding those with discrepant values comprised Set C. The complete set of Hansen et al. was used as Set D. Set E represents a second pharmaceutically relevant set of marketed pharmaceuticals extracted from a recent review by Brambilla and Martelli [44]. The complete Kazius set, Set F, was included in the analysis to give a combined collection of 9423 molecules. A basic summary of the sets is shown in Table 1.

### 2.2 Computational studies

For all PLS and random forest models, a set of 185 2D descriptors available in the MOE software program [45] and a circular Morgan fingerprint [46,47] generated with a radius of 3 bonds (ECFP6) hashed to 1024 count variables using RDKit [48]. Quantum mechanics reaction energies for nitrenium formation from the primary amine were calculated as described in a previous publication considering conformation, tautomer, and spin state [2] using B3LYP hybrid density functional theory energies with a 6-31G* basis set for all C, F, H, O, S, N, and P atoms and the LANL2DZ basis set and ECP for Cl, Br, and I in Gaussian03 [49]. The nitrenium formation energy is equal to the energy of the lowest energy amine conformation subtracted from the energy of the lowest energy nitrenium ion plus hydride anion (ArNH_2 _→ ArNH:^+ ^+ H^-^). The anion formation energy and radical formation energy were similarly calculated and generated and also used the 6-31G* basis set, though improvement in the energy could be expected by adding a diffuse function for the anion. The AM1 [50] HOMO and LUMO orbital energies were calculated using the MOPAC [51] module implemented in MOE. Nitrenium ion charges were determined by using the lowest energy B3LYP/6-31G* nitrenium ion conformation and calculating the NBO population analysis [52,53] using B3LYP and a 6-311G* basis set in Gaussian03.

The random forest classification models used in this article were constructed using the randomForest package [54] for R [55] using the approach developed by Breiman [54,56]. The method was used by constructing 500 unpruned trees using a random sample of sqrt(*N*) of the available predictors for each tree and a 0.632 bootstrap sample of the data for each tree. The remaining data was predicted using the tree and averaged to create the combined out-of-bag (OOB) predictions depicted in the receiver operator characteristic (ROC) plots.

The PLS classifications were done using a PLS regression implemented using the kernel algorithm available in the PLS [57] package in R [55]. Variables showing little variance among cases were removed using the nearZeroVar function in the caret [58] package and all variables were centered by the mean and divided by the standard deviation using the preProcess function in the caret package. The response variable was 0 for Ames- or 1 for Ames+ in these models and the predicted value found from the regression was used as a cutoff in constructing a classification model. All ROC plots and area-under-the-curve (AUC) metrics used the ROCR package [59] in R. Averaging of model performances in the ROC plots was done with vertical averaging of performance at a given false-positive rate, and error bars give the standard deviation. A random sample of 70% of the data was used for training and the process was repeated 100 times representing in part how small batches of Ames results might perform. Variables with zero variance were removed prior to training thus removing 906 variables for the Novartis set and 956 for Set B, and variables were mean-centered and variance-scaled at each training step.

The aryl-amine data sets were constructed as previously described [2]. The all-substructure sets were combined using Pipeline Pilot [60] ignoring chirality due to a lack of chirality in our 2D descriptors and after generating a canonical tautomer. It is also worth noting that absolute chirality determination cannot be done for all compounds and inevitable data entry errors can make this another source of error. A consensus Ames result was used in these all-substructure data with the definition that any Ames+ result in any of the sources was an Ames+ result. Substructure counts were calculated using a Pipeline Pilot [60] protocol with substructure queries that were able to closely reproduce the counts generated in the work of Kazius et al. [21] for their data set (see Additional file 1, Figure S1 and Figure S2 for queries and comparison to this reference). Molecules with molecular weight greater than 700 g/mol were excluded from analysis, which were more than 1.5x outside the interquartile region (IQR). The queries used are provided as Additional file 2. The TOPKAT Ames mutagenicity classification model in the Accelrys ADMET component collection in Pipeline Pilot was used for commercial model predictions. All public data (Sets B, D, E, and F) are provided as a merged sd file as Additional file 3.

The Self-Organizing Map [61] for the combined all-substructure set was generated in Schrodinger Canvas version 1.4 [62] with a 30 cell by 30 hexagonal cell output grid. The program uses Euclidean distance to measure similarity between compounds, and the internal Morgan[46]-type circular fingerprints [47,63] generated with radius 2 and functional atom types were used as descriptors (ECFP4). The TopKat mutagenicity prediction was centered and scaled to give results from 0 to 1 and the random forest model provided probabilities between 0 and 1 for the Ames+ class. The deviation was then the difference between either 1 for an experimentally Ames+ or 0 for Ames- result and the model output. For the aryl-amine set, the 'kohonen' package [64] in R was used instead due to a discovered problem in Canvas with applying trained maps to new compounds. In this case, RDKit was used to generate circular Morgan fingerprints hashed to 1024 count variables as described for the statistical modeling.

## 3. Results and Discussion

In the following results, the differences in the sets are examined in terms of their properties, presence of previously identified mutagenic substructures, and structural similarity and clustering visualized using Kohonen self-organized maps. The difference in predictivity of multiple statistical methods and descriptors between pharmaceutically relevant data and literature compilations is analyzed firstly for aryl-amines and then for sets containing all substructures. For aryl-amines, the quantum mechanically derived reaction energy for forming a known reactive intermediate was shown to be a more stable and accurate predictor than statistical models with more descriptors.

### 3.1 Comparison of molecules in external Ames data sets and pharmaceutically relevant sets

As can be seen in Table 1, the Novartis Set A of aryl-amines and Set C of all substructures tested have a low number of Ames+ compounds compared to their literature counterparts (Sets B and D). In aryl-amines (Set A), only 22% of the molecules are Ames+, and in the entire set of test results at Novartis (Set D), only 15% of the molecules are Ames+. This low percentage is quite similar to other recent reports on Ames results at other pharmaceutical companies such as the recent report from Hillebrecht et al. from Roche [4] where 300/2335 = 13% of the internal compounds were Ames+. A paper by Leach et al. [3] on aryl-amines from AstraZeneca had a slightly higher percentage (109/312 = 35%) of Ames+ aryl-amines less than 250 g/mol. However, in the literature sets (Sets B and D): 71% of aryl-amines and 54% of the entire chemical space are Ames+. Perhaps surprisingly, the marketed pharmaceutical set, set E, has a non-zero incidence of Ames+ test results but it is fairly low-around 12%. An Ames+ test result is only part of a potential drug's profile but the risk of carcinogenicity in later stage animal testing and added regulatory scrutiny present a significant hurdle to drug development in a competitive space.

**Figure 1:**
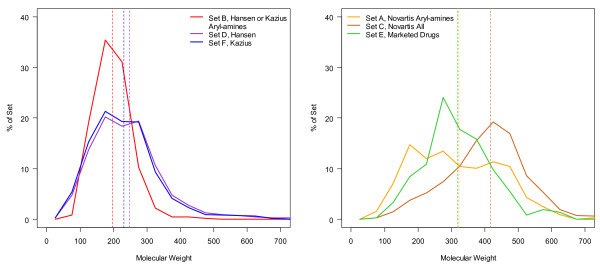
**Molecular weight distributions of Ames test data sets**. Molecular weight distributions of all six sets plotted up to molecular weight of 700 g/mol with mean molecular weight marked with the dotted line of the same color. The literature sets are shown in the left plot, and the Novartis (Sets A and C) and the marketed drugs compilation (Set E) are shown on the right.

Another major difference between the sets is the number of compounds of intermediate molecular weight (200-500 g/mol). This range was nearly absent in the benchmark sets shown in the left plot of Figure 1, but for the Novartis and marketed drugs sets in the right plot, there is a large percentage of the compounds. The bias towards larger molecules likely reflects that the Ames test has often been considered later in drug development, when molecules and their precursors have more complex structures. For all Novartis compounds tested, the median weight was 415 with a fairly wide distribution from 80 to 600 g/mol as shown in Figure 1 (right). In contrast, the median weight for Set D is about 229, with a slightly sharper distribution as shown in the left plot in Figure 1. In quantitative terms, the range of 400-600 g/mol in the literature set, Set D, contains just 372 molecules (6% of the set) compared to 1380 compounds (50% of the set) found in the Novartis Set D. The set of marketed pharmaceuticals with Ames test results is shown in green in the right plot of Figure 1. It has a molecular weight distribution more like the Novartis set, with a median weight of 309 g/mol.

For the aryl-amine sets, Sets A and B, the situation is similar: the Novartis set, Set A, has a higher average molecular weight but there is an even distribution of weights from about 150-500 g/mol. In Set B, there are only 3 aryl-amine data in the range of 400-600 g/mol. In Set A, there are 93, which is almost 30% of the set. The fact that there is such an even distribution, including a large fraction of lower molecular weight compounds, in the Novartis set may reflect the importance of this class and the response to the issue of genotoxicity. When an issue is identified, the typical medicinal chemistry approach is to synthesize dozens of molecules and test all of them. Building blocks that are components of larger molecules are often tested in case of trace genotoxic impurities and for internal guidelines are tested if used for a final clinical candidate. Also drugs for different disease areas such as neuroscience may require smaller molecules.

**Figure 2:**
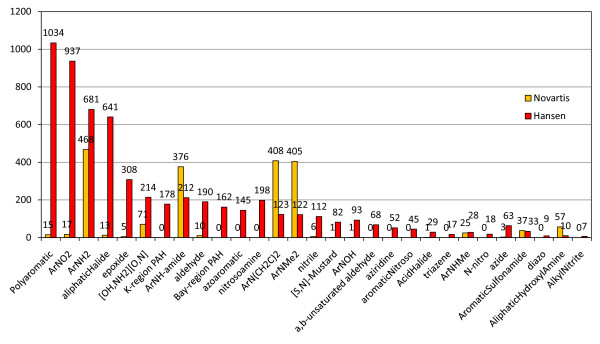
**Mutagenic substructure distributions of Ames test data sets (non-aryl-amine)**. Comparison of Novartis Set C (orange bars) and the Hansen et al. set, Set D (red bars) by mutagenic substructure counts taken from Kazius et al.

The "toxicophores" described in Kazius were used to construct a further comparison of two of the all-substructure sets, Set C (Novartis) and Set D (Hansen). Figure 2 portrays the overall count of these functional groups in the two sets and Table 2 summarizes the percentage of Ames+ compounds in each class, done in a filtered manner where nitroaromatic is the first class. The labels "[OH, NH2][O, N]" and "ArN(CH2C)2", denote an alcohol or amine bonded to an oxygen or nitrogen, such as a hydrazine or a hydroxylamine, and a di-alkylarylamine respectively. Naturally, a number of these functional groups are less common in drug design because of their reactivity or under-represented in test results or in the compounds synthesized due to concerns for toxicity in the Ames test. Aryl-amines, aryl-amine-amides, and dimethylarylamines are quite-well represented and have a lower Ames+ rate. Nitroaromatics were not nearly as represented in this set and are well-established as having a high probability of being responsible for genotoxicity. Building statistical models in the other data sets may benefit greatly from having a feature so strongly associated with genotoxicity. The structures in our set with a nitroaromatic group were Ames+ 40% of the time and in Set D, 84% were Ames+.

**Table 2:** Prevalence of the Kazius toxicophores in Sets C and D.

Functional group	Nitro		ArNH_2_		ArNHC(O)R		ArNHMe		ArNMe_2_		aryl-amine alkylring		ArNHSO_2_Me		unspecified	
**Set**	**C**	**D**	**C**	**D**	**C**	**D**	**C**	**D**	**C**	**D**	**C**	**D**	**C**	**D**	**C**	**D**

# of molecules (sequential filter)	18	973	466	562	401	195	13	22	11	53	274	28	28	13	1523	4660
% of total Set	1%	15%	17%	9%	15%	3%	0%	0%	0%	1%	10%	0%	1%	0%	55%	72%

# Ames+	8	808	91	387	45	77	1	17	3	33	49	13	1	2	162	2160
% Ames+	44%	83%	20%	69%	11%	39%	8%	77%	27%	62%	18%	46%	4%	15%	11%	46%

The % of total set is computed by sequentially filtering by substructure from left to right in the table such that a nitroaromatic molecule is labeled "Nitro" even if it has an aromatic amine substructure "ArNH2".

**Figure 3:**
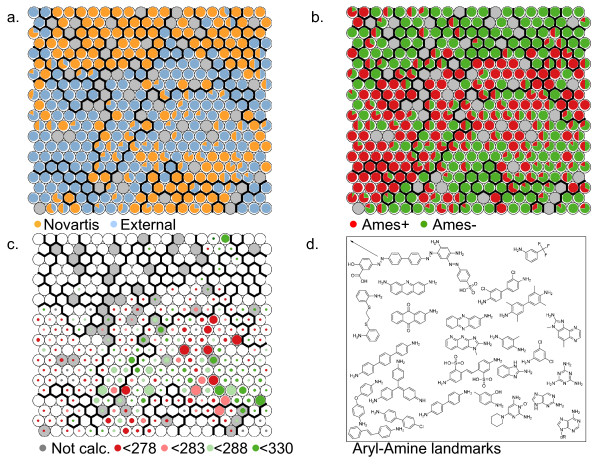
**Self-organizing map of aryl-amine chemical space**. Comparison of aryl-amines in Set C and D using a self-organizing map (SOM) based on circular Morgan fingerprints, the SOM cells are shown in the top two plots with coloring applied based on a.) whether the compound is from Novartis, or b.) whether an Ames+ result (red) exists for the molecules in the cells. In c.) commercial aryl-amines have been mapped to the SOM trained on known aryl-amines and colored by their predicted Ames test result based on nitrenium formation energy in kcal/mol. Size of the marker conveys the number of compounds in the cell. In d.), an approximate location of some Set D aryl-amines is given.

Even within a distinct substructure, aryl-amines, the pharmaceutically relevant set is much different from the Ames test results typically presented in the literature. The use of Kohonen, or Self-Organizing, Maps [61] (SOMs) was helpful for visualizing the differences between the sets using distances between molecular fingerprints of the molecules. This technique clusters molecules with similar substructure with each other in the best matching cell while also maintaining a 2-dimensional grid of cells such that similar molecules appear in adjacent cells. Multidimensional scaling and simple clustering was also investigated for visualization but yielded unsatisfactory neighbors in the first case, and a less useful visualization tool in the second. A SOM map built with the aryl-amines found in all sets is shown in Figure 3 but colored by property. The left plot is colored by where the aryl-amine is from: whether the molecule is a Novartis aryl-amine (orange) or from the external sets (blue). The center plot is colored by whether the compound is Ames+ (red) or Ames- (green). Finally, some representative structures are shown in the approximate locations of the map in the right plot. Cells with some of each class are colored as pie charts depicting the relative fraction of each class present. The approach knows nothing of the set membership of each compound, yet it shows a striking separation of the 1005 aryl-amines both by whether they are part of a drug company's tested compounds or from a literature Ames compilation. Due to the clustering by substructure and that Set D is so largely Ames+, this method also provides excellent separation of the Ames+ aryl-amines. Polyaromatic amines such as aminoacridines, aminophenazines, or aminochrysenes are not highly common in medicinal chemistry. However, they are quite common in the available literature sets. These structures are in Ames+ cells and can be easily represented using molecular fingerprints found in many QSAR models. This makes these sets easier to model.

In Figure 3, we also show where commercial aryl-amines that have been calculated by our model lie in the map. A significant population exists near CF_3_-substituted anilines in the top right, which have historically been Ames- (2^nd ^plot) and have higher nitrenium formation energies. The top left of the map contains mostly larger and more polar aryl-amines, which were purposely left out of the calculations because of the goal of identifying safer starting materials and the better performance of the predictor for lower molecular weight aryl-amines. The center-right area of the map is where a large proportion of the commercially available aryl-amines are located avoiding some of the larger polyaromatic and triphenyl systems. It is also an area that has cells that contain Ames+ and Ames- amines. The nitrenium formation energy predictor can clarify which compounds in this area are safer bets as discussed in the next section.

**Figure 4:**
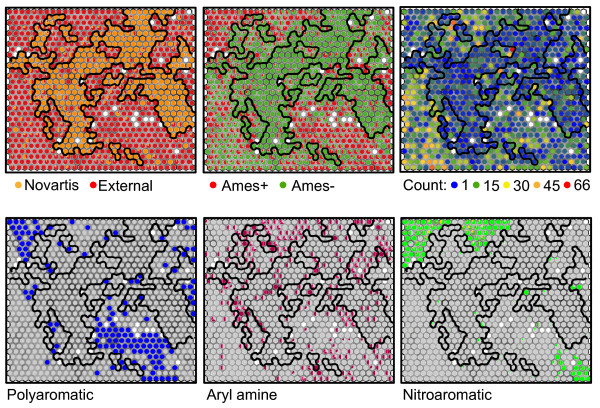
**Self organizing map of the chemical space of compounds considered colored by properties**. SOM for all compounds in Sets C, D, E, and F colored according to property (with pie charts to represent the percentage of molecules in the cell matching a property). The properties labeled from top-left clockwise: whether the compound is in the Novartis deck, whether it has an Ames+ result, the number of molecules in each cell, whether the polyaromatic substructure occurs in a cell, how many compounds have an aryl-amine substructure present, or how many compounds have a nitroaromatic substructure.

The set of 9423 unique compounds included in sets C, D, E, and F are depicted in the SOM in Figure 4. The left-most, top plot colors the SOM by whether it is from Novartis (orange) or an external set (red), the center-top plot shows the distribution of Ames+ (red) and Ames- (green) compounds, and the upper-right plot shows the population of the cells. For the aryl-amine SOM, the population was somewhat uniform, but in the all-substructure plot, the number of molecules per cell varies from 1 to 66. This is natural due to the more extensive differences in the set. The bottom three plots then further characterize where certain substructures are distributed in the SOM. The blue cells show the presence of a polyaromatic substructure in the bottom-left. The aryl-amines are distributed throughout the area and depicted in shades of red. Those molecules with multiple aryl-amine substructures have an increasingly pink hue sector of the pie marker. Finally in the bottom-right plot, the nitroaromatics are highlighted in shades of green. As in the case of the aryl-amines, multiple substructures are given as separate pie-chart sectors of increasing brightness. These are seen almost solely in the external set and in regions of high mutagenicity.

### 3.2 Predicting aryl-amine mutagenicity using quantum mechanically derived descriptors alone

**Figure 5:**
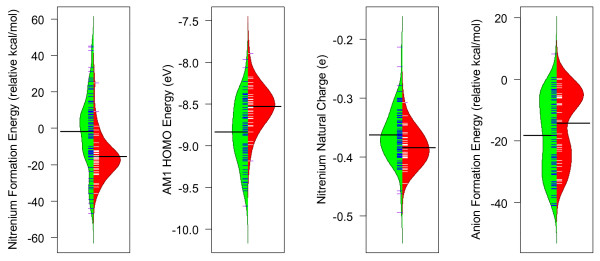
**Beanplots of four QM descriptors considered in our study**. The beanplot is a way to show all data while also conveying a sense of the distribution. In this case, the Ames- points are plotted to the left of the center of each of the four plots with a green distribution and dark points; Ames+ data points are plotted to the right of the center in each plot and shown as white whiskers on a red distribution plot. The mean of each distribution is given as a long dark line. Reaction energies are given relative to aniline.

In a previous report, we determined that for aryl-amines, a case in which reactivity is a principle mode of toxicity, a quantum mechanics reaction energy provides an excellent classifier of Ames+ and Ames- compounds across multiple aryl-amine data sets [2]. The principle of reactivity has been included in models using energies of the HOMO and LUMO orbitals calculated with a fast semi-empirical quantum method such as AM1 or PM3 [34,65,66]. The HOMO energy correlates with the ionization potential, or the energetic cost of losing an electron, while the LUMO correlates to electron affinity, or the gain of an electron. Good performance using these descriptors has been achieved for small sets of aryl-amines with only a few terms in linear classification and regression models [35]. The HOMO energy also does surprisingly well in discriminating Ames+ amines (Figure 5) despite the fact that it does not represent a reaction.

**Figure 6:**
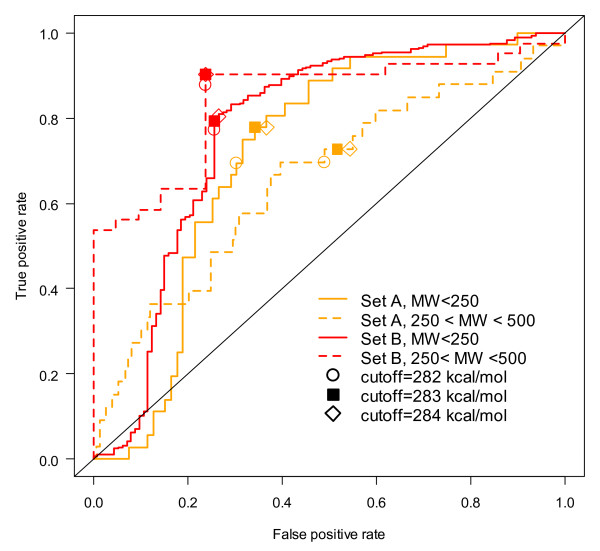
**ROC curve for using the single parameter nitrenium formation energy for aryl-amine sets A and B**. Using the reaction energy for nitrenium intermediate formation (RNH_2 _→ RNH:^+ ^+ H^-^) as a cutoff (labeled by shapes as indicated in the legend) for Novartis and External aryl-amines for the subsets with MW < 250 g/mol (solid line) and 250 < MW < 500 g/mol (dashed line).

A number of groups have also studied the utility of studying the reactions of aryl-amines to understand mutagenicity [2,3,35,67,68]. It was determined that the most statistically significant factor for predicting Ames toxicity was the reaction energy for forming the reactive intermediate, the nitrenium ion, from the aryl-amine [2,3]. This simple descriptor alone can provide a useful prediction of mutagenicity [3,67,68]. These energies are dependent on 3D conformation and the electronic spin state of the reactive intermediate and thus require care to ensure the calculated value is accurate. Using this reaction energy for all Novartis aryl-amines was initially disappointing since good to excellent performance was observed in previous reports for other datasets, in addition to our prediction of external sets gathered for our testing. Upon closer examination, it was clear that most of the sets did not have a uniform distribution up to the range of molecular weight of final pharmaceutical compounds and natural products that comprise a significant portion of the Novartis set. As shown in Figure 6, the performance was much lower for molecules with higher molecular weight in Set A (orange dotted line). Considering that the principle toxicity mechanism of aryl-amines requires metabolic activation, one possible explanation is that larger molecules have more selectivity in metabolic enzymes. Anecdotally, smaller molecular fragments that present themselves as impurities, degradation products or metabolic products were the most common aryl-amine Ames problem at Novartis. Therefore, prediction of lower molecular weight, reagent-like aryl-amines were the principal interest.

Other groups have introduced other quantum mechanics descriptors for aryl-amines in addition to nitrenium forming reactions [2,3,67-70], including the charge on the nitrenium ion nitrogen [68], relative energy of anion formation and relative iron complexation energy in a CYP1A2 binding site model [70], and finally reaction energy for aminyl radical formation, another species that could be produced in the cytochrome systems and has been associated with DNA damage [71]. Some of the reactions that have been used are summarized below in Equations 1-5 and these have been compared for Set A. The Pearson correlation matrix in Table 3 shows that all of the nitrenium forming processes represented by Equations 1-3 are closely correlated and all provide good discrimination. The area-under-the-curve (AUC) for the ROC plot for each of these parameters is given in Table 3 and shown graphically in Additional file 1 (Figure S3).

**Table 3:** Correlation matrix of quantum mechanics parameters for Set A and the single-parameter ROC AUC performance.

	**1**	**2**	**3**	**4**	**5**	**6**	**7**	**8**	**9**	**10**	**AUC**
	
1. NitFormE (Eq. 1)	1	1.0	1.0	0.8	-0.2	-0.8	-0.3	0.3	0.9	-0.3	0.74
2. NitFormE (Eq. 2)	1.0	1	1.0	0.8	-0.2	-0.8	-0.3	0.3	0.9	-0.3	0.73
3. NitFormE (Eq. 3)	1.0	1.0	1	0.7	-0.2	-0.8	-0.3	0.3	0.9	-0.3	0.73
4. RadicalFormE (Eq. 4)	0.8	0.8	0.7	1	0.0	-0.5	-0.2	0.2	0.7	-0.3	0.68
5. Anion Form. E. (Eq. 5)	-0.2	-0.2	-0.2	0.0	1	0.4	0.8	0.6	0.1	0.2	0.60
6. AM1 HOMO E	-0.8	-0.8	-0.8	-0.5	0.4	1	0.5	-0.3	-0.7	0.4	0.74
7. AM1 LUMO E	-0.3	-0.3	-0.3	-0.2	0.8	0.5	1	0.7	-0.1	0.2	0.60
8. HOMO-LUMO E	0.3	0.3	0.3	0.2	0.6	-0.3	0.7	1	0.5	-0.1	0.56
9. Nitrenium Charge	0.9	0.9	0.9	0.7	0.1	-0.7	-0.1	0.5	1	-0.2	0.66
10. Expermental Ames (Ames+ = 1)	-0.3	-0.3	-0.3	-0.3	0.2	0.4	0.2	-0.1	-0.2	1	

See Additional file 1, Figure S3 for a graphical representation of the ROC curves.

The best single parameters include the nitrenium formation energy reactions and the AM1 HOMO orbital energy. The Ames+ compounds have values tightly clustered in these two parameters as shown in the beanplots in Figure 5 and show the expected relationship to the barrier of forming the nitrenium intermediate. Larger HOMO orbital energies of the amine and lower reaction energies for forming the reactive nitrenium ion would make it easier to form the intermediate. Ames+ amines tend to have a more negative charge on the nitrenium nitrogen, which has been presented previously [68], but the relationship is clearly not as strong. As suggested in a recent article [70], we looked at the anion formation energy (Equation 5) and though on its own it has little discrimination as shown in Figure 5 and its AUC in Table 3, it appears to provide a useful complement to the nitrenium formation energy. Higher sensitivity at equivalent false-positive ratios in the 80-87% sensitivity region of the ROC curve (Additional file 1, Figure S3) were possible. A PLS model using all of the quantum mechanical descriptors showed a large loading value in the first component for nitrenium formation energies and the anion formation energy had the largest loading value in the second component. The starting geometries for the anions can be generated using the same procedure for generating the nitrenium ions from the B3LYP-optimized aryl-amines.

Out of all of the QM parameters, the most useful parameter by PLS loadings and Random Forest variable importance (top ranked in all runs) using all of the data was the nitrenium formation energy (Equation 1). This particular reaction is also the easiest to calculate out of Equations 1-3 since it reduces the number of atoms in the system compared to losing -OH or -OAc as the leaving group (Equations 2 and 3). While HOMO energy has a high correlation with nitrenium formation energy (0.84), the nitrenium formation energy provided better overall performance in the 70-84% sensitivity range.

(1) $$\text{ArN}{\text{H}}_{\text{2}}\to \text{ArNH}{:}^{+}{+}^{-}\text{H}$$

(2) $$\text{ArNOAc}\to \text{ArNH}{:}^{+}+\;-\text{OAc}$$

(3) $$\text{ArNOH }+\;{\text{H}}_{\text{3}}{\text{O}}^{+}\to \text{ArNH}{:}^{+}+\text{ 2 }{\text{H}}_{\text{2}}\text{O}$$

(4) $$\text{ArN}{\text{H}}_{\text{2}}\to \text{ArN}\text{H}\begin{array}{c} . \end{array}+\;\text{H}\begin{array}{c} . \end{array}$$

(5) $$\text{ArN}{\text{H}}_{\text{2}}+\;{\text{H}}_{\text{2}}\text{O}\to \text{ArNH}-+\;{\text{H}}_{\text{3}}{\text{O}}^{+}$$

### 3.3 Predicting aryl-amine Ames test results using multivariate statistics

Multi-dimensional statistical models improving upon the performance of the nitrenium formation energy parameter alone were difficult to construct. A number of available approaches including *k*-Nearest-Neighbors (*k*NN), random forest, partial least squares (PLS), support vector machines (SVM), and PLS with discriminant analysis provided similar performance for Set A. A comparison of these methods and other approaches to modeling Ames toxicity when all mutagens are included have already been presented in other studies [36]. We have chosen to focus on PLS and random forest analysis of the aryl-amine data for further discussion because of the interpretability of PLS, the ability to include a large number of correlated variables, and the straightforward assessment of the importance of variables.

**Figure 7:**
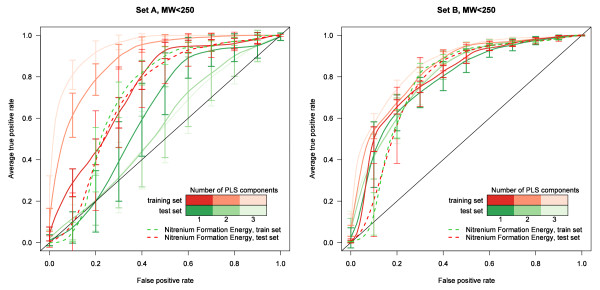
**Averaged ROC curves of PLS models for aryl-amine sets (MW < 250 g/mol)**. Performance using 1, 2, or 3 components using 100 randomly sampled test and training sets is shown with Set A on the left and Set B on right. The darker shades of lines have fewer components (see scale) and the green ROC curves are for the 100 random test sets (30% sample) and the red for 100 training sets (remaining 70% of set) both averaged over identical false positive rates. Error bars represent standard deviation.

Figure 7 shows the receiver operator characteristic (ROC) curve averaged over true-positive rates for 100 PLS models at identical false-positive rates for Set A (left) and Set B (right) using 1, 2, and 3 components of a PLS model. As summarized in Table 4, the performance of the method on the Novartis set, Set A, was highly variable and significantly poorer than for Set B. The performance on the test set decreased dramatically when adding a second component leading to a decrease in average AUC of 0.12. The same approach for the external set, Set B, resulted in a significantly higher AUC performance in the test set of 0.79, 0.80, and 0.81 for a 1-, 2-, or 3-component PLS model respectively. This was higher than the test set performance of Set A by 0.17 for the PLS models in the test set. After 2 components, the test set accuracy at a cutoff chosen in the training set to produce 80% sensitivity began to decrease for Set B. The random forest out-of-bag model performance on the test set for Set B averaged over 100 runs was significantly better than the 2-component PLS model. The performance of a random forest model for Set A was almost identical to the 1-component PLS model, so the AUC performance difference between Set A and Set B random forest models was an even higher 0.24.

**Table 4:** Performance of prediction methods.

Model		Set A, Training	Set A, Test	Set B, Training	Set B, Test
PLS with NitFormE	PC1	0.76 ± 0.02	0.63 ± 0.08	0.80 ± 0.02	0.78 ± 0.05
	PC2	0.88 ± 0.03	0.56 ± 0.09	0.83 ± 0.01	0.80 ± 0.04

PLS without NitFormE	PC1	0.76 ± 0.02	0.62 ± 0.08	0.80 ± 0.02	0.79 ± 0.05
	PC2	0.89 ± 0.02	0.54 ± 0.09	0.84 ± 0.01	0.80 ± 0.04

Nitrenium Formation Energy Alone		0.72 ± 0.04	0.71 ± 0.09	0.78 ± 0.02	0.77 ± 0.05

Random Forest with NitFormE			0.62 ± 0.01		0.855 ± 0.003
Random Forest without Nitrenium Formation Energy			0.61 ± 0.01		0.851 ± 0.003

A single-parameter nitrenium formation energy as a model is compared to PLS and random forest models with and without the nitrenium formation energy parameter.

For both Set A and Set B, the multiple-variable PLS models offered an improved prediction over using nitrenium formation energy alone (dashed line) in the training set but not in the test set. The performance of the Set A PLS model on the test set was much worse on average than using this single parameter. The model in Set B was slightly better but unfortunately, most of the performance increase over the nitrenium formation energy (0.03 in AUC) was in a low-sensitivity region of the ROC curve (< 50% true positive rate). The prediction in this range was not considered to be useful for excluding Ames+ fragments. These results are frustrating but provoked thought about why the molecules commonly used in the literature are different and easier to model.

In an attempt to address the problem of overfitting in this PLS model, a smaller selection of variables was chosen guided by the PLS loading weights, Pearson correlation between variables, and variable importances from a random forest model of the set. The weights were averaged over the 100 models and the largest 30 mean loading weights were used. Table 5 shows the variable loading and jack-knife significance testing run in the PLS cross-validation as well as the mean decrease in Gini coefficient over all trees for the random forest model built with the widest selection of parameters. Two additional descriptors (the Balaban j index [72] and density) are given, which were suggested by random forest importance measures and their low correlation with the other descriptors. The Balaban index was also identified as a discriminating variable in a previous investigation of aryl-amines and depends partly on the number of rings [3].

**Table 5:** Selected variables important in statistical models of Set A and Set B.

Variable	Set A PC1	Mean Decrease in Gini	log *p*	Set B PC1	Set B PC2	Mean Decrease in Gini	log *p*
**chi0v_C**	**0.153 (1)**	**0.4 (30)**	**-5**	**0.148 (2)**	**-0.070 (86)**	**1.5 (17)**	**-8**
**GCUT_SLOGP_3**	**0.151 (4)**	**1.07 (1)**	**-4**	**0.141 (5)**	**-0.059 (102)**	**2.3 (8)**	**-8**
**a_count**	**0.152 (2)**	**0.3 (62)**	**-6**	**0.129 (19)**	**-0.108 (51)**	**1 (54)**	**-2**
**balabanJ**	**-0.097 (60)**	**0.5 (15)**	**-2**	**-0.128 (20)**	**0.004 (241)**	**2.6 (7)**	**-8**
**a_nH**	**0.149 (5)**	**0.4 (40)**	**-4**	**0.115 (35)**	**-0.077 (76)**	**1 (52)**	**-1**
**Density**	**-0.108 (51)**	**0.4 (32)**	**-2**	**-0.106 (47)**	**-0.058 (103)**	**2.7 (6)**	**-7**
**NitFormE**	**-0.125 (26)**	**1.0 (2)**	**-3**	**-0.104 (52)**	**-0.04 (136)**	**8.9 (1)**	**-8**
**Q_VSA_POS**	**0.129 (24)**	**0.6 (7)**	**-3**	**0.061 (87)**	**-0.052 (108)**	**1.4 (26)**	**-1**
**BCUT_SMR_3**	**0.124 (28)**	**0.6 (9)**	**-2**	**0.046 (111)**	**-0.129 (32)**	**1.4 (24)**	**-1**
*a_nC*	*0.146 (10)*	*0.3 (57)*	*-4*	*0.148 (3)*	*-0.073 (80)*	*0.8 (74)*	*-12*
*Morgan0672*	*0.056 (111)*	*0.1 (130)*	*0*	*0.107 (44)*	*-0.045 (123)*	*0.3 (144)*	*-3*
*a_hyd*	*0.140 (15)*	*0.2 (121)*	*-3*	*0.143 (4)*	*-0.065 (94)*	*0.5 (114)*	*-5*
b_1rotN	0.075 (82)	0.05 (187)	-1	0.001 (244)	-0.154 (11)	0.3 (146)	-4
KierFlex	0.085 (73)	0.3 (69)	0	-0.011 (206)	-0.184 (4)	1.6 (14)	-7
b_rotN	0.065 (97)	0.03 (217)	-1	-0.004 (232)	-0.16 (8)	0.3 (146)	-5
Morgan0280	-0.042 (147)	0.02 (242)	-1	-0.06 (88)	-0.149 (14)	0.2 (160)	-8
a_nO	-0.04 (150)	0.03 (223)	0	-0.073 (75)	-0.139 (25)	0.3 (148)	-8

PLS loadings, the PLS jackknife significance estimate from leave-one-out cross-validation, and the random forest mean decrease in Gini coefficient for each variable is given below with their overall rank by that measure. All variable abbreviations follow those in MOE. Bolded, italicized, and underlined text respectively indicates variables chosen to be important in both Set A and Set B from the first principal component, important for Set B in the first component, and important for Set B in the second component.

The first principal component included the nitrenium formation energy and other descriptors relating to electrostatics, hydrophobicity, and indirect properties such as the number of atoms. The variable chi0v_C (^0^χ_C_), [73] is a valence-modified carbon atom connectivity index which depends on the number of carbon atoms in the structure and how many non-hydrogen atoms are connected to them. a_count and a_nH are simply the number of atoms and hydrogens respectively. GCUT_SLOGP calculates log P based on atomic contributions and a modified graph distance [74], while BCUT_SMR calculates the molar refractivity based on atomic contributions and bond order [75-77]. Q_VSA_POS is the sum of atomic contributions to van der Waals surface area where the sum of partial charges of the atoms are positive [78], and density is the molecular weight divided by total van der Waals volume.

The most interpretable variables in the second component for Set B related to flexibility and included the number of rotatable bonds and number of rotatable single-bonds, b_rotN and b_1rotN respectively, the Kier flexibility parameter [79], abbreviated here as KierFlex. The number of oxygen atoms and a fingerprint bit associated with an aryl-amine substructure was also significant.

**Figure 8:**
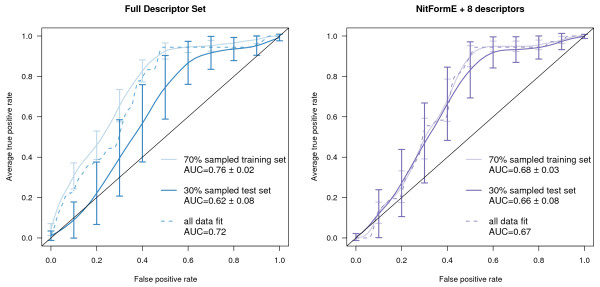
**Averaged ROC curves for Set A aryl-amine PLS models**. The plot on the left is for the model built with a full descriptor set and the plot on the right is for a limited descriptor set. The left plot shows the averaged (identical false positive values) ROC curves for the test (dark line) and training (lighter line) sets of 100 PLS models built on a random 70% sample of the Set A aryl-amine data (MW < 250 g/mol) and the performance of a PLS model built on all of the data as a dashed line with all non-zero-variance descriptors. The right plot uses only 9 descriptors including nitrenium formation energy.

**Figure 9:**
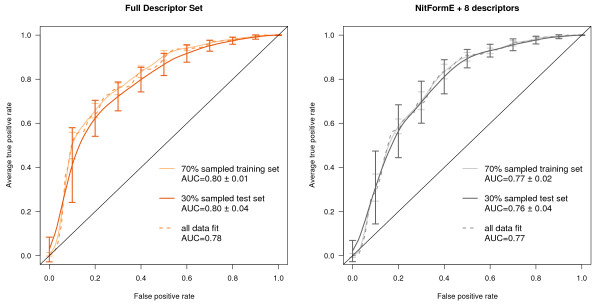
**Averaged ROC curves for Set B aryl-amine PLS models**. The plot on the left uses a full descriptor set while the plot on the right is for a model using a limited descriptor set. The left plot shows the averaged (identical false positive values) ROC curves for the test (dark line) and training (lighter line) sets of 100 PLS models built on a random 70% sample of the Set B aryl-amine data (MW < 250 g/mol) and the performance of a PLS model built on all of the data as a dashed line with all non-zero-variance descriptors. The right plot uses only 9 descriptors including nitrenium formation energy.

Using just the first component parameters shown in bold in Table 5 resulted in less decrease in performance between training and test sets and decreased performance by less than 0.03 AUC. Fitting all data led to an intermediate performance between the training and test sets as would be expected. A random forest model using only these descriptors performed much better than one using all of the potential descriptors for Set A, and for Set B this approach had similar but slightly lower performance. The likely overfitting in the random forest model was quite surprising and indicates a tendency for many of the parameters to introduce conflicting results. These results are summarized in Table 6 and the full ROC curves are shown in Figures 8 and 9. The single parameter nitrenium formation energy can met or exceeded the performance of PLS models that were given far more information. It was also able to perform well on the challenging Novartis set.

**Table 6:** Performance of PLS and random forest models on the aryl-amine sets.

		Set A, Training	Set A, Test	Set B, Training	Set B, Test
PLS with NitFormE	PC1	0.76 ± 0.02	0.63 ± 0.08	0.80 ± 0.02	0.78 ± 0.05
PLS with 9 descriptors	PC1	0.68 ± 0.03	0.66 ± 0.08	0.77 ± 0.02	0.76 ± 0.04

Random Forest With NitFormE			0.62 ± 0.01		0.855 ± 0.003
Random Forest With 9 descriptors			0.682 ± 0.008		0.844 ± 0.004

Nitrenium Formation Energy		0.72 ± 0.04	0.71 ± 0.09	0.78 ± 0.02	0.77 ± 0.05

The performance of statistical models for Set A and Set B, over 100 random samples of the data. For the random forest model, the performance is for the data when it was out-of-bag in the construction of the trees and for PLS, it is for a 30% test sample when the model is trained with the other 70%.

### 3.4 Cross set performance-training with Set A and testing Set B and vice versa

**Figure 10:**
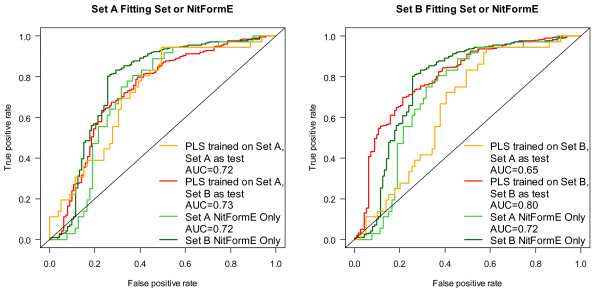
**Extrapolation from one aryl-amine data set to another: cross-set performance**. Comparison of the performance obtained when a PLS (1-component) model is fitted using all of the data in Set A and used to predict Set B (left plot, solid red line) to that obtained when fitting to the data in Set B and using that model to predict the data in Set A (right plot, solid orange line). The performance for the training set is also shown in dashed lines and the performance of using only the nitrenium formation energy is shown with a dot-dashed line.

**Figure 11:**
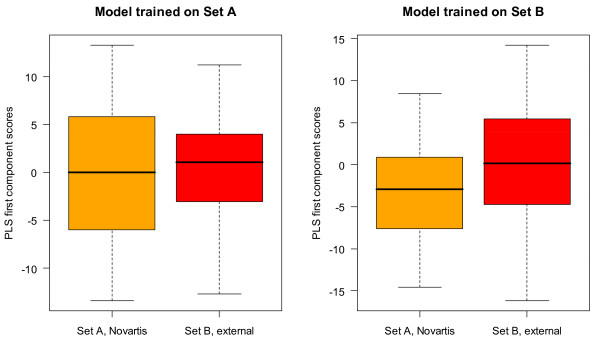
**Extrapolation from one aryl-amine data set to another: cross-set Tukey Boxplot of the distribution of scores**. Distribution of scores obtained for Set A (orange box) and Set B (red box) when applying a model trained on all of the data in Set A (left) or a model trained on all of the data in Set B (right) as a measure of outliers and domain.

In a further attempt to characterize the differences in Set A and Set B, the sets were used as a test set for a model built from the other set. The results of this experiment are shown in Table 7 and Figure 10. The difference in performance was quite instructive and shows that the performance of Set B is less able to extrapolate to the aryl-amines in Set A than vice versa. The performance of the Set A model was actually better for Set B data than for the data used to train it while a model based on Set B had clear difficulty in predicting Set A. This can be quickly seen in Figure 10 which shows the ROC curve for Set B predicted by a PLS model built on Set A (solid red line, left graph) and the ROC curve for Set A predicted by Set B (solid orange line, right graph). The performance of the Set A model on Set B (0.73) was almost identical by AUC to the Set A performance (0.72), representing a decrease in AUC of 0.07 from the Set B model performance. In fact even the 9-descriptor Set A model gave a performance of 0.73 for Set B. PLS models built on Set B performed extremely well on Set B with AUCs around 0.8. However, when these models were applied to Set A, the performance was markedly worse and the 9-descriptor model performed much better than the model with all of the descriptors. The unscaled PLS scores are shown in Figure 11 in the form of a boxplot for each model. The Set A scores are broadly and almost normally distributed in the Set A model encompassing all of the scores of the Set B data, mostly in the middle 50% of the data (interquartile range). However, the Set B data is not as centered in the model and most of the Set A data is outside the middle 50% of the Set B scores.

**Table 7:** Performance for PLS and random forest models for the other set.

	Set A		Set B
Trained on 100% Set A			

PLS 1-component	0.72	→	0.73
PLS 1-component, 9 descriptors	0.67	→	0.73
Random Forest	0.68*	→	0.72

Trained on 100% Set B			

PLS 1-component all descriptors	0.65	←	0.80
PLS 1-component 9 descriptors	0.68	←	0.77
Random Forest	0.71	←	0.84*

All of Set A or Set B is used as the training set, and the other is used as the test set.

*performance when out-of-bag

### 3.5 Performance of a commercial model on aryl-amine data-TOPKAT

**Figure 12:**
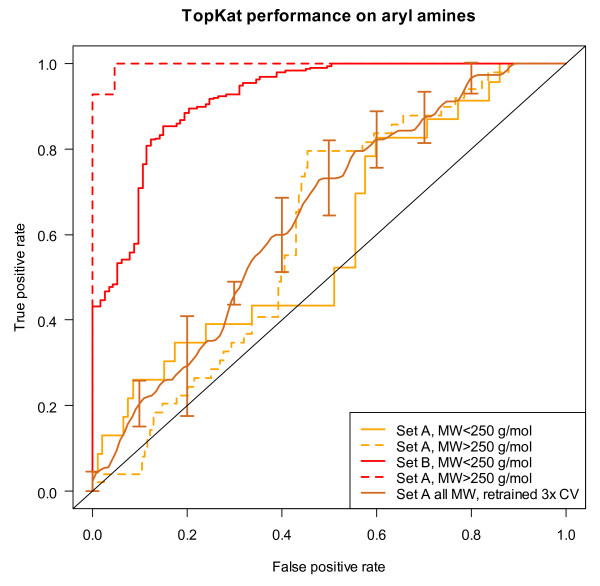
**Performance of the TopKat Ames mutagenicity prediction module on aryl-amines**. Performance of the default, prebuilt TopKat Ames mutagenicity model on Set A (orange) and Set B (red) for MW < 250 g/mol (solid line) or for 250 < MW < 500 g/mol (dashed). Additionally, the vertically averaged performance of a 3-fold random cross-validated retraining of the TopKat model using Set A is shown in brown with standard deviation error bars.

The pre-built mutagenicity prediction model available to us in TopKat [80] was explored as a possible prediction method. The performance of the prebuilt model on the 327 aryl-amines in Set A was quite poor with an AUC of the ROC curve of 0.59 for the molecular weight < 250 g/mol subset and 0.61 for the molecular weight ≥ 250 g/mol subset. The model provides the Tanimoto similarity with the most similar compound used to construct the model as one way to assess model applicability. Set A has an average closest Tanimoto distance of 0.41 ± 0.14 for aryl-amines less than 250 g/mol molecular weight and 0.57 ± 0.07 for those between 250 and 500 g/mol. At least a large portion of the aryl-amine data in Set B was used to build the TOPKAT model and the aryl-amines in this set have an average Tanimoto distance close to zero and a fantastic AUC performance of 0.92 for MW < 250 g/mol (*N *= 398) and 0.997 for MW ≥ 250 (*N *= 62). Although it could be argued that these models require retraining when applied to data far from the training set, such data are often not available. A simple retraining using a three-fold cross-validation experiment, resulted in only marginal improvement in performance for the Novartis set with AUCs 0.60, 0.64, and 0.69 achieved in the three test sets over the AUCs of 0.58, 0.63, and 0.57 obtained for the default model for the respective sets. Most of the improvement in the ROC curve was in the range of more than 50% false positive performance, which would not be considered a useful range. The ROC curves for these investigations are shown in Figure 12. The good performance for the aryl-amines in Set B suggests that the aryl-amine substructure alone is not problematic in developing these models. Previous publications have not separated the performance by substructure, so it was unclear that this would be true.

### 3.6 Modeling Ames test results for all substructures

Given the difficulty of addressing aryl-amines, we began to search for reasons the set would be more difficult and if the result would be true for more than just this subspace. Literature reports have provided excellent results for benchmark sets containing all mutagens and small collections of aryl-amines or nitroaromatics. Even better performance could be obtained using multiple models based on the applicability domain of a mutagen under consideration such as Sushko et al. [40] for multiple substructures and Leong et al. [41] for just the aryl-amine substructure. Though surveys of the poor performance of pre-built commercial model performance on proprietary sets has been presented, reports on models of large proprietary sets and delineation of substructure seemed to be lacking. A classification model given a collection of distinct features strongly associated with mutagenicity would be expected to perform better than a model missing such clear-cut mutagenic features such as nitroaromatics mentioned previously.

**Figure 13:**
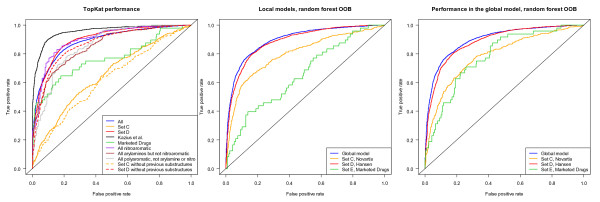
**ROC curve performance for models on all-substructure data sets**. The left plot shows the performance of the default TopKat Ames mutagenicity model on Sets C (orange), D (red), E (green), and F (black) as well as the performance for particular substructures in all sets (blue): nitroaromatics (purple), aryl-amines (brown), or polyaromatic (gray). Dotted lines for Sets C and D show performance after removing these substructures. The center plot shows the out-of-bag performance of random forest models built on Sets C (orange), D (red), and E (green) and the global model (blue) when they are used as the training set. The right plot shows the Set C, D, or E subsets of the global out-of-bag performance (blue) of a random forest model built on all of the data.

Table 8 and Figure 13 describes the performance of 2 global models, the TopKat pre-built commercial model and a random forest model built from all data in Sets C, D, E, and F. For clarity, substructure ROC plot performance is shown only for the TopKat in the left plot. Removing molecules with the typically mutagenic polyaromatic, aryl-amine, and nitroaromatic substructures resulted in significant performance decreases in both models in both Set C (Novartis, orange, solid line to orange, dashed line) and Set D (Hansen et al., red, solid line to red, dashed line). The decreases in performance were greater for the TopKat model and for Set D. The global random forest model contained more training data which improved the performance on Set D compared to TopKat, and Set C had fewer of these mutagenic substructures as was presented in Figure 2. The nitroaromatic mutagenic substructure had much better performance in the TopKat model and accounts for over 10% of Set D. However, the nitroaromatic subset in the random forest global model and the aryl-amine and polyaromatic mutagenic substructure performance in both models were equivalent or slightly worse than the overall performance.

**Table 8:** Performance of models on subsets of the compiled all-substructure set.

Molecule set	**Random Forest**^**a**^	TopKat	**Local**^**b, c**^	Local (remaining data)
Set C	0.79	0.65	0.80	0.65
Not polyaromatic, ArNH_2_, ArNO_2_	0.78	0.62	-	

Set D	0.88	0.90	0.89	0.69
Not polyaromatic, ArNH_2_, ArNO_2_	0.86	0.86	-	

Set E	0.78	0.77	0.66	0.67

Kazius et al.	0.91	0.95	-	

All	0.90^b^	0.89	-	
Nitroaromatics	0.85	0.90	-	
Aryl-amines (not nitroaromatic or polyaromatic)	0.87	0.87	-	
Polyaromatic (not nitroaromatic)	0.75	0.86	-	

The four columns denote a random forest model built on the full set of data, the default TopKat[76,77] model, a local random forest models built only on the indicated set, and the performance of this local model on the rest of the compiled all-substructure set.

^a^Global model, trained on all data, ^b^OOB performance, ^c^Trained only on the particular set

**Figure 14:**
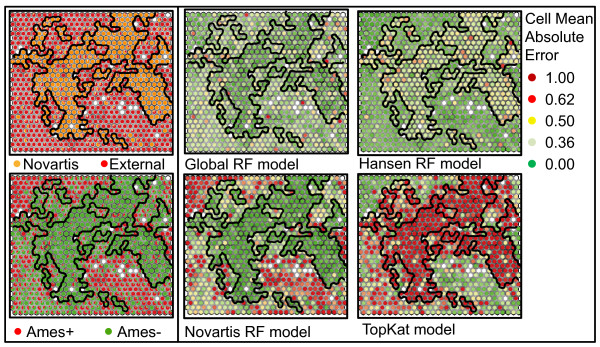
**Performance of global models mapped to chemical space**. Self-organizing map of Sets C, D, E, and F (from Figure 4) again colored by whether the molecule is in the Novartis set (Set C, orange) or is Ames+ (red) in the left box. In the right box, the SOM is colored by the mean absolute error of the predictions in the cell for the indicated model. Dark green indicates correct classification, yellow an equivocal prediction, and red an incorrect prediction. Cell mean absolute error is defined as the difference between the predicted probability of being mutagenic and the experimental class (0 or 1).

The performance of the random forest model built on the global set was similar to the performance of local random forest models built on the individual sets for Sets C and D. It is important to note the extremely high performance for the Kazius set which is a large portion of the training data in the TopKat method. The random forest model constructed from all of the data also did well on this set which again indicates that it is inherently a simpler set to model using the commonly used descriptors. This all-data model has good performance across the entire chemical space map as detailed in Figure 14 where greens indicate a successful prediction (over 500 trees) while yellow indicates an equivocal prediction and oranges and reds would be expected to give the wrong prediction. In fact, a random forest model built with just Set D provided a fairly good prediction but gave more equivocal results in the regions occupied mainly by Novartis compounds. As might be expected from the good performance of TopKat on set D and poor performance on the Novartis set, Figure 14 shows that most of the cells with Novartis compounds would be misclassified by the TopKat model (red cells) but also gets other regions wrong such as the lower left-hand corner. Unlike the case of the aryl-amines, the Novartis all-substructure model does not perform well on regions occupied mostly by the other sets. The performance map provides almost a perfect opposite to TopKat, though the lower-left-hand corner is still difficult to predict with many equivocal cells. Looking at the Ames+/Ames- coloring of the cells in the left-hand plot gives an idea of why this might be possible. This region of substructure space has many cells that contain a mix of Ames+ and Ames- compounds.

## 4. Conclusions

In this article we have shown that there are significant differences in the physicochemical and biological properties of compounds used in drug discovery and those in compiled Ames test results from the literature. This includes molecular weight, substructure distribution, and the percentage of mutagenic compounds in the data set. This is important to communicate, as much of the literature data is being used to test prediction methods as well as playing a role in current testing strategy debates. The compounds in the Novartis test results are mostly drug precursor molecules, while literature mutagenicity results are often petrochemicals and pesticides of primary concern as environmental pollutants. The size and complexity of the molecules tested at Novartis was significantly larger on average than that of molecules included in external sets, as visualized by distributions in molecular weight. Chemical functional groups or substructures that have a high association with mutagenicity determined from the literature data are largely absent in the Novartis set taking away a valuable discrimination feature. Additionally, the proportion of mutagens in external sets is higher and disturbingly close to 50% as might occur from successive culling for balanced model development. As a result of these factors, many drug discovery molecules are outside the applicability domain of pre-built commercial models. The data is also more difficult due to lack of strongly associated structural features and would lead to worse performance of these statistical models if they were included in the training set. Therefore these models cannot provide adequate performance to predict, let alone, avoid a positive Ames test. The Ames test, as well as other genotoxicity tests, continue to be a significant problem in drug discovery, and companies should work together to share data with the wider community of scientists and organizations. The best-validated and best-performing prediction available for low molecular weight aryl-amines is still a quantum-mechanics reaction energy representing the formation of the nitrenium ion. Effective predictive models could be built for all-substructure sets using the random forest methodology and commonly available 2D descriptors and chemical fingerprints. Performance was still significantly lower for molecules from Novartis and marketed pharmaceuticals. Despite extensive work in the area of predicting this particular toxicity, work in designing more difficult test sets and more adaptable models is still necessary.

## Abbreviations

Ames+: positive Ames test result; Ames-: negative Ames test result; PLS: partial least squares; ROC: receiver operator characteristic; AUC: area-under-the-curve; N: number of points; NitFormE: nitrenium formation energy; SOM: self-organizing map; OOB: out-of-bag; HOMO: highest occupied molecular orbital; LUMO: lowest occupied molecular orbital; IQR: interquartile range

## Competing interests

The authors are employees of a pharmaceutical company and must comply with regulatory guidelines for genotoxicity; however, all analysis is based on the 2-strain non-GLP screening version of the test. Novartis licenses software for a fee from Schrodinger, Accelrys, CCG, and Gaussian, Inc.

## Authors' contributions

PM wrote, developed the idea behind the work, and performed the statistical analysis in the paper. CS substantially contributed to the idea for the paper, discussion, statistical experiments, editing, and technique suggestions. LW contributed significantly to the idea for the paper, guidance, editing, and discussion. All authors have reviewed and approved the final manuscript.

## Supplementary Material

Additional file 1 **Supplemental figures and tables**. Supplemental figures and tables referred to in the text of the article. Click here for file

Additional file 2 **Ames toxicophore queries**. Compressed library of Ames toxicophore queries as.mol files. Click here for file

Additional file 3 **Public structures used in the research**. SDF file with 6812 structures and data in public sets B, D, E, and F of the paper. Fields BrambillaMarketedDrug, Hansen, and Kazius indicate which set the structure is in and if this is 1, the corresponding fields BrambillaExpAmes, HansenExpAmes, or KaziusExpAmes respectively will indicate the Ames result for that set. Sets D, E, and F are unfiltered, all-substructure sets: Set D is from Hansen et al.,[38] Set E is the marketed drug set taken from Brambilla et al. [44], and Set F is from Kazius et al.[21] Set B is an aryl amine set which includes the union of Set D and F filtered by the aryl amine substructure. Other fields are results of counting the query substructures in Additional file 1. Click here for file

## References

[B1] Mirza A Desai R Reynisson J Known drug space as a metric in exploring the boundaries of drug-like chemical space Eur J Med Chem 2009 44 5006 5011 10.1016/j.ejmech.2009.08.014 19782440

[B2] McCarren P Bebernitz GR Gedeck P Glowienke S Grondine MS Kirman LC Klickstein J Schuster HF Whitehead L Avoidance of the Ames test liability for arylamines via computation Bioorg & Med Chem 2011 19 3173 3182 10.1016/j.bmc.2011.03.066 21524589

[B3] Leach AG Cann R Tomasi S Reaction energies computed with density functional theory correspond with a whole organism effect; modelling the Ames test for mutagenicity Chem Commun 2009 1094 1096 10.1039/b818744d 19225647

[B4] Hillebrecht A Muster W Brigo A Kansy M Weiser T Singer T Comparative Evaluation of in Silico Systems for Ames Test Mutagenicity Prediction: Scope and Limitations Chem Res Toxicol 2011 24 843 854 10.1021/tx2000398 21534561

[B5] Naven RT Louise-May S Greene N The computational prediction of genotoxicity Epxert Opin Drug Metab Toxicol 2010 6 797 807 10.1517/17425255.2010.495118 20528613

[B6] Waldron HA A brief history of scrotal cancer Br J Ind Med 1983 40 390 401 10.1136/oem.40.4.390 PMC1009212 6354246

[B7] S2(R1) Guidance on genotoxicity testing and data interpretation for pharmaceuticals intended for human use 2008 Geneva, Switzerland: ICH 28 22675782

[B8] Fetterman BA Kim BS Margolin BH Schildcrout JS Smith MG Wagner SM Zeiger E Predicting rodent carcinogenicity from mutagenic potency measured in the Ames Salmonella assay Environ Mol Mutagen 1997 29 312 322 10.1002/(sici)1098-2280(1997)29:3<312::aid-em12>3.0.co;2-h 9142175

[B9] Benigni R Bossa C Tcheremenskaia O Giuliani A Alternatives to the carcinogenicity bioassay: in silico methods, and the in vitro and in vivo mutagenicity assays Epxert Opin Drug Metab Toxic 2010 6 809 819 10.1517/17425255.2010.486400 20438313

[B10] Mortelmans K Zeiger E The Ames Salmonella/microsome mutagenicity assay Mutat Res-Fundam Mol Mech Mutag 2000 455 29 60 10.1016/s0027-5107(00)00064-6 11113466

[B11] McCann J Choi E Yamasaki E Ames BN Detection of carcinogens as mutagens in the Salmonella/microsome test: assay of 300 chemicals Proc Natl Acad Sci 1975 72 USA 5135 5139 10.1073/pnas.72.12.5135 PMC388891 1061098

[B12] Mortelmans K Isolation of plasmid pKM101 in the Stocker laboratory Mutat Res-Rev Mutat 2006 612 151 164 10.1016/j.mrrev.2006.03.002 16716644

[B13] Diehl MS Willaby SL Snyder RD Comparison of the Results of a Modifed Miniscreen and the Standard Bacterial Reverse Mutation Assays Environ Mol Mutagen 2000 35 72 77 10.1002/1098-2280(2000)36:1<72::aid-em10>3.0.co;2-y 10918362

[B14] Knight AW Little S Houck K Dix D Judson R Richard A McCarroll N Akerman G Yang C Birrell L Walmsley RM Evaluation of high-throughput genotoxicity assays used in profiling the US EPA ToxCast (TM) chemicals Regul Toxicol Pharm 2009 55 188 199 10.1016/j.yrtph.2009.07.004 19591892

[B15] Westerink WMA Stevenson JCR Lauwers A Griffioen G Horbach GJ Schoonen WGEJ Evaluation of the Vitotox(TM) and RadarScreen assays for the rapid assessment of genotoxicity in the early research phase of drug development Mutagenesis Mutat Res-Genet Toxicol Environ Mutag 2009 676 113 130 10.1016/j.mrgentox.2009.04.008 19393335

[B16] Matthews EJ Kruhlak NL Cimino MC Benz RD Contrera JF An analysis of genetic toxicity, reproductive and developmental toxicity, and carcinogenicity data: I. Identification of carcinogens using surrogate endpoints Regul Toxicol Pharm 2006 44 83 96 10.1016/j.yrtph.2005.11.003 16386343

[B17] Hansen K Stern RM A survey of metal-induced Mutagenicity in vitro and in vivo Toxicol Environ Chem 1984 9 87 91

[B18] Kenyon MO Cheung JR Dobo KL Ku WW An evaluation of the sensitivity of the Ames assay to discern low-level mutagenic impurities Regul Toxicol Pharm 2007 48 75 86 10.1016/j.yrtph.2007.01.006 17379368

[B19] Looker AR Ryan MP Neubert-Langille BJ Naji R Risk Assessment of Potentially Genotoxic Impurities within the Framework of Quality by Design Org Process Res Dev 2010 14 1032 1036

[B20] Loeb LA Harris CC Advances in chemical carcinogenesis: a historical review and prospective Cancer Res 2008 68 6863 6872 10.1158/0008-5472.CAN-08-2852 PMC2583449 18757397

[B21] Kazius J McGuire R Bursi R Derivation and Validation of Toxicophores for Mutagenicity Prediction J Med Chem 2005 48 312 320 10.1021/jm040835a 15634026

[B22] Sanderson DM Earnshaw CG Computer Prediction of Possible Toxic Action from Chemical Structure; The DEREK System Hum Exp Toxicol 1991 10 261 273 10.1177/096032719101000405 1679649

[B23] Miller JA Carcinogenesis by chemicals: an overview-GHA Clowes memorial lecture Cancer Res 1970 30 559 4915745

[B24] Skipper PL Kim MY Sun HLP Wogan GN Tannenbaum SR Monocyclic aromatic amines as potential human carcinogens: old is new again Carcinogenesis 2010 31 50 58 10.1093/carcin/bgp267 PMC2802674 19887514

[B25] Hillier SM Marquis JC Zayas B Wishnok JS Liberman RG Skipper PL Tannenbaum SR Essigmann JM Croy RG DNA adducts formed by a novel antitumor agent 11β-dichloro in vitro and in vivo Mol Cancer Ther 2006 5 977 984 10.1158/1535-7163.MCT-05-0464 16648569

[B26] Ridings JE Barratt MD Cary R Earnshaw CG Eggington CE Ellis MK Judson PN Langowski JJ Marchant CA Payne MP Watson WP Yih TD Computer prediction of possible toxic action from chemical structure: an update on the DEREK system Toxicology 1996 106 267 279 10.1016/0300-483x(95)03190-q 8571398

[B27] Ashby J Fundamental structural alerts to potential carcinogenicity or noncarcinogenicity Environ Mutagen 1985 7 919 921 10.1002/em.2860070613 4065064

[B28] Ashby J Tennant RW Definitive relationships among chemical structure, carcinogenicity and mutagenicity for 301 chemicals tested by the U.S. NTP Mutat Res 1991 257 229 306 10.1016/0165-1110(91)90003-e 1707500

[B29] Kalgutkar AS Gardner I Obach RS Shaffer CL Callegari E Henne KR Mutlib AE Dalvie DK Lee JS Nakai Y O'Donnell JP Boer J Harriman SP A Comprehensive Listing of Bioactivation Pathways of Organic Functional Groups Curr Drug Metab 2005 6 161 225 10.2174/1389200054021799 15975040

[B30] Snyder R Pearl G Mandakas G Choy W Goodsaid F Rosenblum I Assessment of the sensitivity of the computational programs DEREK, TOPKAT, and MCASE in the prediction of the genotoxicity of pharmaceutical molecules Environ Mol Mutagen 2004 43 143 158 10.1002/em.20013 15065202

[B31] Snyder R Smith M Computational prediction of genotoxicity: room for improvement Drug Discov Today 2005 10 1119 1124 10.1016/S1359-6446(05)03505-1 16182197

[B32] Glowienke S S18: In silico assessment of safety concerns esp. of carcino-genic potential Exp Toxicol Pathol 2009 61 264 265

[B33] Benigni R Bossa C Predictivity and Reliability of QSAR Models: The Case of Mutagens and Carcinogens Toxicol Mech 2008 18 137 147 10.1080/15376510701857056 20020910

[B34] Debnath AK Debnath G Shusterman AJ Hansch C A QSAR investigation of the role of hydrophobicity in regulating mutagenicity in the ames test: 1. Mutagenicity of aromatic and heteroaromatic amines inSalmonella typhimurium TA98 and TA100 Environ Mol Mutagen 1992 19 37 52 10.1002/em.2850190107 1732103

[B35] Hatch FT Colvin ME Quantitative structure-activity relationships of mutagenic aromatic and heterocyclic amines Mutat Res-Fundam Mol Mech Mutag 1997 376 87 96 10.1016/s0027-5107(97)00029-8 9202742

[B36] Hansch C Structure-activity relationships of chemical mutagens and carcinogens Total Environ 1991 109-110 17 29 10.1016/0048-9697(91)90167-d 1815351

[B37] Zhang Q-Y Aires-de-Sousa J Random Forest Prediction of Mutagenicity from Empirical Physicochemical Descriptors J Chem Inf Model 2006 47 1 8 10.1021/ci050520j 17238242

[B38] Hansen K Mika S Schroeter T Sutter A ter Laak A Steger-Hartmann T Heinrich N Müller K-R Benchmark Data Set for in Silico Prediction of Ames Mutagenicity J Chem Inf Model 2009 49 2077 2081 10.1021/ci900161g 19702240

[B39] Benigni R Bossa C Netzeva T Worth A Collection and Evaluation of (Q)SAR Models for Mutagenicity and Carcinogenicity 2007 Luxembourg: Office for Official Publications of the European Communities 119

[B40] Sushko I Novotarskyi S Körner R Pandey AK Cherkasov A Li J Gramatica P Hansen K Schroeter T Müller K-R Xi L Liu H Yao X Öberg T Hormozdiari F Dao P Sahinalp C Todeschini R Polishchuk P Artemenko A Kuz'min V Martin TM Young DM Fourches D Muratov E Tropsha A Baskin I Horvath D Marcou G Muller C Varnek A Prokopenko VV Tetko IV Applicability Domains for Classification Problems: Benchmarking of Distance to Models for Ames Mutagenicity Set J Chem Inf Model 2010 50 2094 2111 10.1021/ci100253r 21033656

[B41] Leong MK Lin S-W Chen H-B Tsai F-Y Predicting Mutagenicity of Aromatic Amines by Various Machine Learning Approaches Toxicol Sci 2010 116 498 513 10.1093/toxsci/kfq159 20507879

[B42] Hardy B Douglas N Helma C Rautenberg M Jeliazkova N Jeliazkov V Nikolova I Benigni R Tcheremenskaia O Kramer S Girschick T Buchwald F Wicker J Karwath A Gütlein M Maunz A Sarimveis H Melagraki G Afantitis A Sopasakis P Gallagher D Poroikov V Filimonov D Zakharov A Lagunin A Gloriozova T Novikov S Skvortsova N Druzhilovsky D Chawla S Ghosh I Ray S Patel H Escher S Collaborative development of predictive toxicology applications CheminformaticsJ Cheminf 2010 2 7 10.1186/1758-2946-2-7 PMC2941473 20807436

[B43] Benchmark Data Set for In Silico Prediction of Ames Mutagenicity Sdf file available at [http://doc.ml.tu-berlin.de/toxbenchmark/], set 2, 6512 compounds 10.1021/ci900161g 19702240

[B44] Brambilla G Martelli A Update on genotoxicity and carcinogenicity testing of 472 marketed pharmaceuticals Mutat Res-Rev Mutat 2009 681 209 229 10.1016/j.mrrev.2008.09.002 18845271

[B45] Molecular Operating Environmentversion 2009.10, Chemical Computing Group, Montreal, Canada

[B46] Morgan HL The Generation of a Unique Machine Description for Chemical Structures-A Technique Developed at Chemical Abstracts Service J Chem Doc 1965 5 107 113

[B47] Rogers D Hahn M Extended-Connectivity Fingerprints J Chem Inf Model 2010 50 742 754 10.1021/ci100050t 20426451

[B48] Landrum G RDKit http://sourceforge.net/projects/rdkit version Q3 2010

[B49] Frisch MJ Trucks GW Schlegel HB Scuseria GE Robb MA Cheeseman JR Montgomery JA Vreven T Kudin KN Burant JC Millam JM Iyengar SS Tomasi J Barone V Mennucci B Cossi M Scalmani G Rega N Petersson GA Nakatsuji H Hada M Ehara M Toyota K Fukuda R Hasegawa J Ishida M Nakajima T Honda Y Kitao O Nakai H Klene M Li X Knox JE Hratchian HP Cross JB Bakken V Adamo C Jaramillo J Gomperts R Stratmann RE Yazyev O Austin AJ Cammi R Pomelli C Ochterski JW Ayala PY Morokuma K Voth GA Salvador P Dannenberg JJ Zakrzewski VG Dapprich S Daniels AD Strain MC Farkas O Malick DK Rabuck AD Raghavachari K Foresman JB Cui JVO Baboul AG Clifford S Cioslowski J Stefanov BB Liu G Liashenko A Piskorz P Komaromi I Martin RL Fox DJ Keith T Al-Laham MA Peng CY Nanayakkara A Challacombe M Gill PMW Johnson B Chen W Wong MW Gonzalez C Pople JA Gaussian 03 2004 version Rev. E.01 Gaussian, Inc.: Wallingford, CT

[B50] Dewar MJS Zoebisch EG Healy EF Stewart JJP Development and use of quantum mechanical molecular models. 76. AM1: a new general purpose quantum mechanical molecular model J Am Chem Soc 1985 107 3902 3909

[B51] MOPAC 1993 version 7.1 Stewart JJP.

[B52] Foster JP Weinhold F Natural hybrid orbitals J Am Chem Soc 1980 102 7211 7218

[B53] Reed A Weinstock R Weinhold F Natural population analysis J Chem Phys 1985 83 735 746

[B54] Liaw A Wiener M Classification and Regression by randomForest R News 2002 2 18 22

[B55] Team RDC R: A Language and Environment for Statistical Computing 2008 version 2.6.2 Vienna, Austria: The R Foundation for Statistical Computing; Vienna, Austria

[B56] Breiman L Random Forests Machine Learning 2001 45 5 32

[B57] pls: Partial Least Squares Regression (PLSR) and Principal Component Regression (PCR) 2007 version 2.1-0 Ron Wehrens and Bjørn-Helge Mevik http://mevik.net/work/software/pls.html

[B58] caret: Classification and Regression Training 2008 version 3.21 Max Kuhn. Contributions from Jed Wing, Steve Weston and Andre Williams http://caret.r-forge.r-project.org/Classification_and_Regression_Training.html

[B59] Sing T Sander O Beerenwinkel N Lengauer T ROCR: visualizing classifier performance in R Bioinformatics 2005 21 3940 3941 10.1093/bioinformatics/bti623 16096348

[B60] Pipeline Pilot 2008 version 7.5 Accelrys Software, Inc.: San Diego, CA 92121

[B61] Kohonen T Self-Organizing Maps 2001 3 Berlin: Springer

[B62] Canvas 2011 version 1.4 Schrodinger, LLC: New York, New York

[B63] Duan J Dixon SL Lowrie JF Sherman W Analysis and comparison of 2D fingerprints: Insights into database screening performance using eight fingerprint methods J Mol Graphics Modell 2010 29 157 170 10.1016/j.jmgm.2010.05.008 20579912

[B64] Wehrens R Buydens LMC Self- and Super-organising Maps in R: the kohonen package J Stat Softw 2007 21

[B65] Benigni R Bossa C Netzeva T Rodomonte A Tsakovska I Mechanistic QSAR of aromatic amines: New models for discriminating between homocyclic mutagens and nonmutagens, and validation of models for carcinogens Environ Mol Mutagen 2007 48 754 771 10.1002/em.20355 18008355

[B66] Benigni R Passerini L Gallo G Giorgi F Cotta-Ramusino M QSAR models for discriminating between mutagenic and nonmutagenic aromatic and heteroaromatic amines Environ Mol Mutagen 1998 32 75 83 10.1002/(sici)1098-2280(1998)32:1<75::aid-em9>3.0.co;2-a 9707101

[B67] Bentzien J Hickey ER Kemper RA Brewer ML An in Silico Method for Predicting Ames Activities of Primary Aromatic Amines by Calculating the Stabilities of Nitrenium Ions J Chem Inf Model 2010 50 274 297 10.1021/ci900378x 20078034

[B68] Borosky GL Ultimate Carcinogenic Metabolites from Aromatic and Heterocyclic Aromatic Amines:A Computational Study in Relation to Their Mutagenic Potency Chem Res Toxicol 2007 20 171 180 10.1021/tx600278q 17261035

[B69] Colvin M Seidl E Nielsen I Le Bui L Hatch F Deprotonation and hydride shifts in nitrenium and iminium forms of aminoimidazole-azaarene mutagens Chem Biol Interact 1997 108 39 66 10.1016/s0009-2797(97)00094-x 9463520

[B70] Shamovsky I Ripa L Börjesson L Mee C Nordén B Hansen P Hasselgren C O'Donovan M Sjö P Explanation for Main Features of Structure-Genotoxicity Relationships of Aromatic Amines by Theoretical Studies of Their Activation Pathways in CYP1A2 J Am Chem Soc 2011 133 16168 16185 10.1021/ja206427u 21894985

[B71] Sarkar FH Radcliff G Callewaert DM Purified prostaglandin synthase activates aromatic amines to derivatives that are mutagenic to Salmonella typhimurium Mutat Res 1992 282 273 281 10.1016/0165-7992(92)90134-4 1379690

[B72] Balaban AT Highly discriminating distance-based topological index Chem Phys Lett 1982 89 399 404

[B73] Kier LB Hall LH Nature of structure-activity-relationships and their relation to molecular connectivity Eur J Med Chem 1977 12 307 312

[B74] Wildman SA Crippen GM Prediction of Physicochemical Parameters by Atomic Contributions Chem Inf Comput Sci 1999 39 868 873

[B75] Pearlman RS Smith KM Metric Validation and the Receptor-Relevant Subspace Concept J Chem Inf Comput Sci 1999 39 28 35

[B76] Burden FR Molecular identification number for substructure searches J Chem Inf Comput Sci 1989 29 225 227

[B77] Burden FR A chemically intuitive molecular index based on the eigenvalues of a modified adjacency matrix Quant Struct-Act Relat 1997 16 309 314

[B78] Gasteiger J Marsili M Iterative partial equalization of orbital electronegativity: a rapid access to atomic charges Tetrahedron 1980 36 3219 3222

[B79] Hall LH Kier LB The molecular connectivity chi indexes and kappa shape indexes in structure-property modeling Rev Comput Chem 1991 2 367 422

[B80] TOPKATversion 6.2 Accelrys: San Diego, CA 92121

