# Supplementary material for: An investigation into pharmaceutically relevant mutagenicity data and the influence on Ames predictive potential
Source: J Cheminform. 2011 Nov 22;3:51. doi: 10.1186/1758-2946-3-51 (PMC3277490; doi:10.1186/1758-2946-3-51)
Supplement: Additional file 1 — Supplemental figures and tables. Supplemental figures and tables referred to in the text of the article. [file 1758-2946-3-51-S1.DOC]

**Supporting Information**

Building models of difficult sets of mutagenicity data based on quantum mechanical description of reactivity

Patrick McCarren^a^, Clayton Springer^a^, and Lewis Whitehead^a^,*

^a^ Novartis Institutes for Biomedical Research, 100 Technology Square, Cambridge, MA 02139, USA.

Table S1. A selection of reported Ames test classification models.

| Reference | Substructures | Model type | Training set accuracy | N | N_ames+ | Ntest | NtestAmes+ | Accuracy | AUC test |
| --- | --- | --- | --- | --- | --- | --- | --- | --- | --- |
| benigni 2007[1] | Aryl amines TA98 | linear regression | 0.89 | 111 | 86 |  |  | 0.69 |  |
| benigni 2008[2] | Aryl amines TA100 | linear regression | 0.87 | 111 | 64 |  |  | 0.81 |  |
| Hansen 2009[3] | All | svm |  | 5525-5528 | 3503 | 984-987 | 570-584 |  | 0.86 |
| Ferrari and Gini 2010[4] | All | svm | 0.90 | 3367 | 1883 | 837 | 451 | 0.81 |  |
| Matthews, Kruhlak,Cimino, Benz, Contrera[5] | All | MC4PC |  |  |  | 1403 | 633 | 0.81 |  |
| Zhang and Aires-deSousa[6] | All | random forest | 0.84 | 4083 | 2308 | 472 | 305 | 0.85 |  |
| Langham and Jain[7] | All | ensemble learning | 0.79 | 4337 | 2401 | 400 | 174 | 0.75 | 0.839 |
| Helma, Cramer,Kramer, deRaedt[8] | All | PART | 0.94 | 684 | 341 | leave10%out | | 0.76 |  |
| Saiakhov[9] | All | MC4PC | 0.79 |  |  | 984-987 | 570-584 | 0.73 |  |


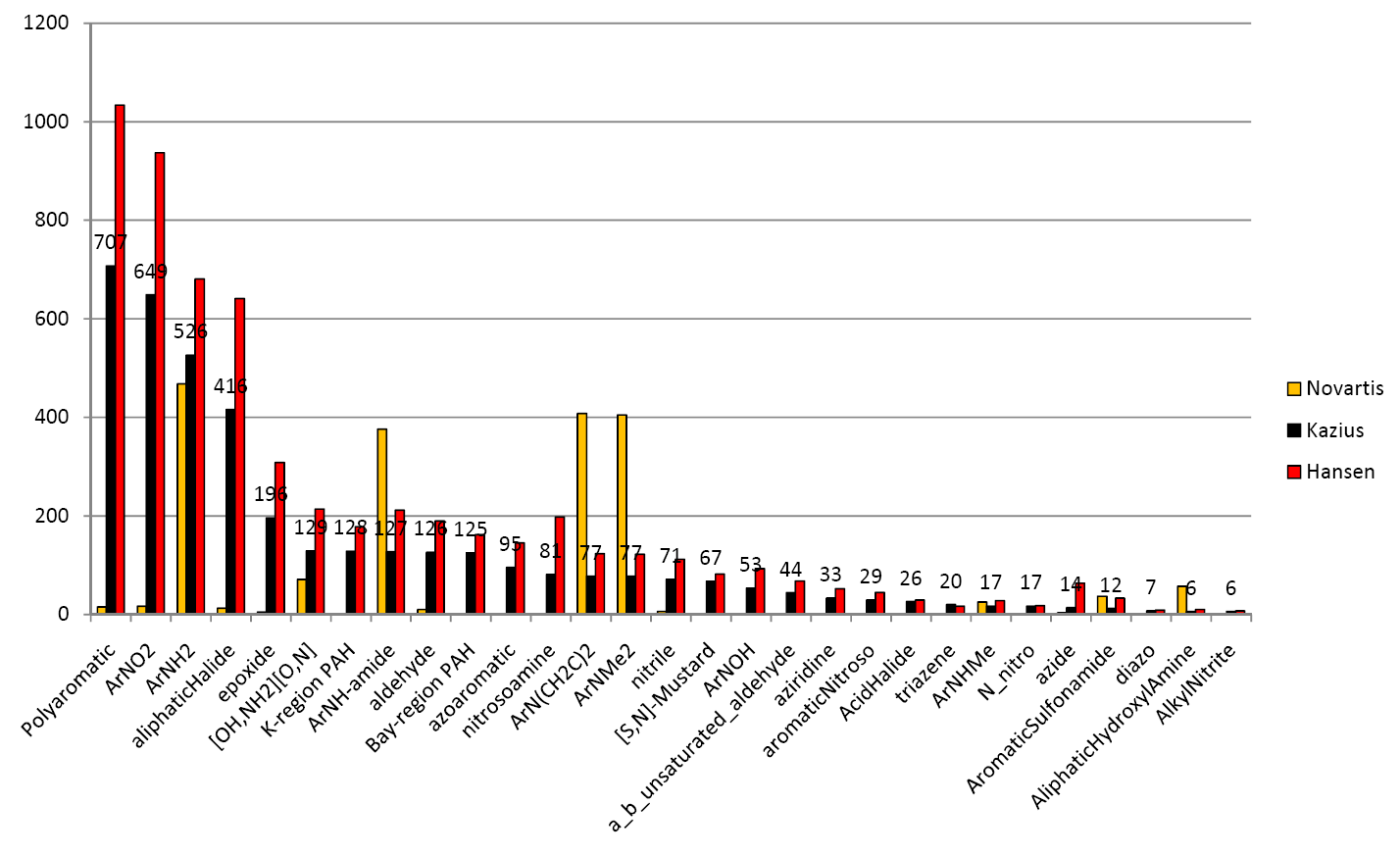


Figure S1. Substructure Counts for Sets C, D, and F full sets.

Table S2. Comparison to Kazius 2005 substructure counts to counts derived from present substructure queries.

| Toxicophore | Kazius count | This work |
| --- | --- | --- |
| aromatic nitro | 644 | 649 |
| aromatic amine | 508 | 526 |
| aromatic nitroso | 32 | 29 |
| alkyl nitrite | 6 | 6 |
| nitrosamine | 80 | 81 |
| epoxide | 196 | 196 |
| aziridine | 33 | 33 |
| azide | 14 | 14 |
| diazo | 7 | 7 |
| triazene | 20 | 20 |
| aromatic azo | 88 | 95 |
| unsubstituted heteroatom-bonded heteroatom | 128 | 129 |
| aromatic hydroxylamine | 53 | 53 |
| aliphatic halide | 416 | 416 |
| carboxylic acid halide | 26 | 26 |
| nitrogen or sulfur mustard | 67 | 67 |
| bay-region in polycyclic aromatic hydrocarbons | 125 | 125 |
| K-region in polycyclic aromatic hydrocarbons | 128 | 128 |
| polycyclic aromatic system | 660 | 707 |

Figure S2. Summary of query structures to construct Figure 2, Figure S1, and Table S2.


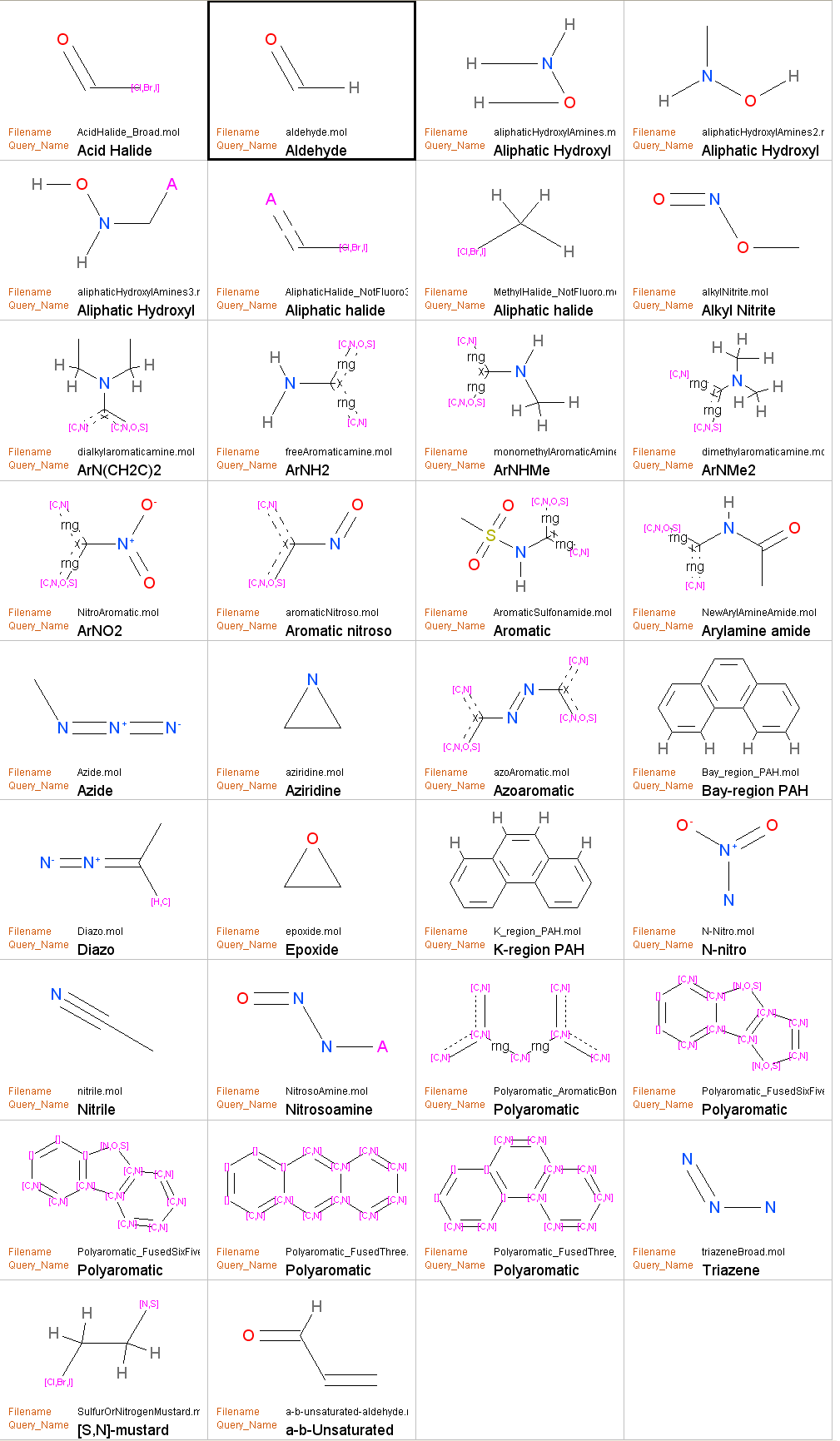


Figure S3. Comparison of performance of all QM descriptors shown in Table 3 and the performance of two PLS models.

References
